# Intravenous delivery of enzalutamide based on high drug loading multifunctional graphene oxide nanoparticles for castration-resistant prostate cancer therapy

**DOI:** 10.1186/s12951-020-00607-4

**Published:** 2020-03-18

**Authors:** Wenjun Jiang, Jiyuan Chen, Chunai Gong, Yuanyuan Wang, Yuan Gao, Yongfang Yuan

**Affiliations:** 1 Department of Clinical Pharmacy and Pharmaceutical Management, School of Pharmacy, Fudan University, 826 Zhangheng Road, Shanghai, 201203 China; 2 Department of Pharmacy, East China University of Science and Technology, 130 Meilong Road, Shanghai, 200237 China; 3 Department of Pharmacy, Shanghai Ninth People’s Hospital, Shanghai Jiao Tong University School of Medicine, 639 Zhizaoju Road, Shanghai, 200011 China; 4 Department of Pharmacy, Changhai Hospital, Second Military Medical University, 168 Changhai Road, Shanghai, 200433 China

**Keywords:** Redox-sensitive, Enzalutamide, Graphene quantum dot derivate, Castration-resistant prostate cancer

## Abstract

**Background:**

Enzalutamide (Enz) has shown limited bioavailability via oral administration. Castration-resistant prostate cancer (CRPC) is frequent among patients receiving 18–24 months of androgen deprivation therapy. The nonsteroidal anti-androgen enzalutamide (Enz) used in the treatment of prostate cancer has shown limited bioavailability via oral administration. Therefore, we developed a multifunctional enzalutamide-loaded graphene oxide nanosystem (TP-GQDss/Enz) for CRPC intravenous treatment, with high drug loading efficiency.

**Methods:**

Aminated graphene quantum dots (GQDs) were first cross-linked via disulfide bonds into a graphene quantum dot derivative of approximately 200 nm (GQDss), which was further functionalized with a tumour-targeting peptide and PEG to form TP-GQDss. Enz was loaded into TP-GQDss for in vitro and in vivo study.

**Results:**

The results showed that high drug-loading efficiency was achieved by TP-GQDss via π–π electron interaction. TP-GQDss could be rapidly internalized by CRPC cells via endocytosis. Moreover, Enz in TP-GQDss could inhibit the growth of C4-2B and LNCaP prostate cancer cell lines in vitro. Further, TP-GQDss exhibited an enhanced cancer-targeting ability and alleviated the side effects of Enz in vivo.

**Conclusions:**

The multifunctional nanocarrier constructed here could accomplish controlled Enz release and serve as an intravenous therapy platform for CRPC.
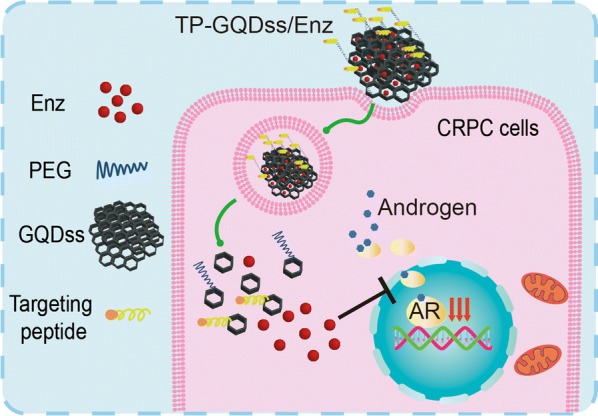

## Background

Over the past few decades, prostate cancer has been one of the most dangerous cancers in men [1]. Until now, androgen deprivation therapy (ADT), which inhibits androgen receptor (AR) signalling, has been the primary treatment for early-stage prostate cancer. However, after 18–24 months of ADT, most patients will eventually develop castration-resistant prostate cancer (CRPC). Enzalutamide (Enz) is a clinically approved second-generation nonsteroidal anti-androgen drug for the treatment of CRPC [2]. It could improve the efficacy of CRPC therapy via competitively inhibiting the interaction between androgen and AR. However, Enz has shown limited bioavailability via oral administration and increased systemic side effects when patients were exposed at high doses [3–6]. Therefore, new strategies to enhance the targeted delivery to reduce systemic side effects and improve bioavailability of Enz are urgently needed.

Nanoparticles have been reported to increase targeting abilities and efficacies in vivo [7]. Our previous studies demonstrated that small molecules or gene therapy drugs in intelligent nanoplatforms could be accurately delivered to prostate cancer, achieving high efficacy in vitro and in vivo [8–10]. Due to the large dose of Enz needed in clinical applications, it is a challenge to intravenously deliver hydrophobic and low permeability drugs such as Enz [11]. Thus, a nano-vehicle with a high drug-loading capacity should be applied for Enz delivery. Graphene oxide (GO) possesses unique characteristics, including good colloidal stability, a tunable surface, high drug loading efficiency and biocompatibility [12]. Aromatic drugs could be effectively absorbed by GO through π–π stacking. The active functional groups on the surface of GO facilitate its surface functionalization. Covalent binding of PEG to GO has been widely used to improve the stability and long circulation of GO with high drug-loading capacity [13, 14]. GO modified by antibodies or peptides or other bioactive molecules could achieve targeted delivery [15–17]. Though GO-based nanocarriers feature a number of advantages over other nanocarriers, they are restricted by their poor drug-release capacity. The cumulative drug-release rate only reaches approximately 40% in 48 h even with near-infrared radiation (NIR) [18]. Additionally, it is hard to prepare GO derivatives with the expected size and hydrophilicity [19].

**Fig. 1:**
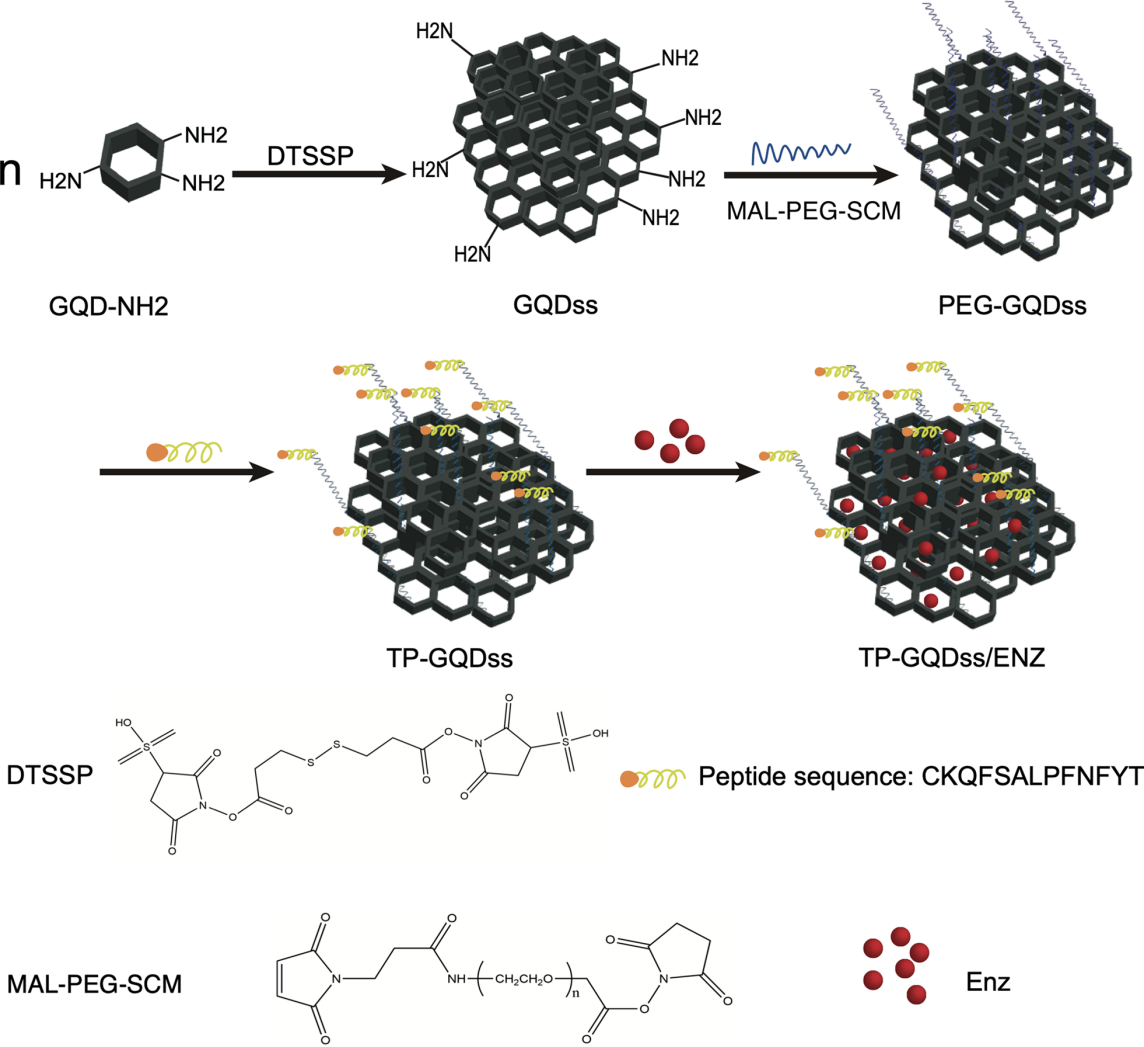
The design and preparation steps for TP-GQDss/Enz

In this study, aminated graphene quantum dots (GQDs-NH_2_) were disulfide cross-linked and modified with PEG as well as targeting peptide (TP-GQDss) for Enz delivery (TP-GQDss/Enz) (Fig. 1). It was reported that TP was specific to prostate cancer and could recognize more than 70% of surgical prostate cancer sections [20]. Here, graphene quantum dots (GQDs) were first cross-linked via disulfide bonds into graphene quantum dot derivatives (GQDss) for Enz delivery. As shown in Fig. 1, GQDs were cross-linked via the redox-sensitive amino crosslinking agent 3,3′-dithiobis(sulfosuccinimidyl propionate) (DTSSP) to obtain GQDss and then modified with PEG and TP. The surface of TP-GQDss was loaded with Enz with the aid of aromatic rings. The system provided the following key features: (1) high drug loading efficiency; (2) controlled release of Enz in the tumour site via a redox-sensitive mechanism; and (3) biocompatibility and efficient targeted delivery by PEG and TP. We hypothesized that Enz in TP-GQDss (TP-GQDss/Enz) might achieve enhanced drug delivery and biocompatibility after intravenous administration. For this purpose, TP-GQDss/Enz was prepared, while the targeted delivery and therapeutic effects as well as its biocompatibility were evaluated on in vitro and in vivo models.

## Results

### Materials characterization

**Fig. 2:**
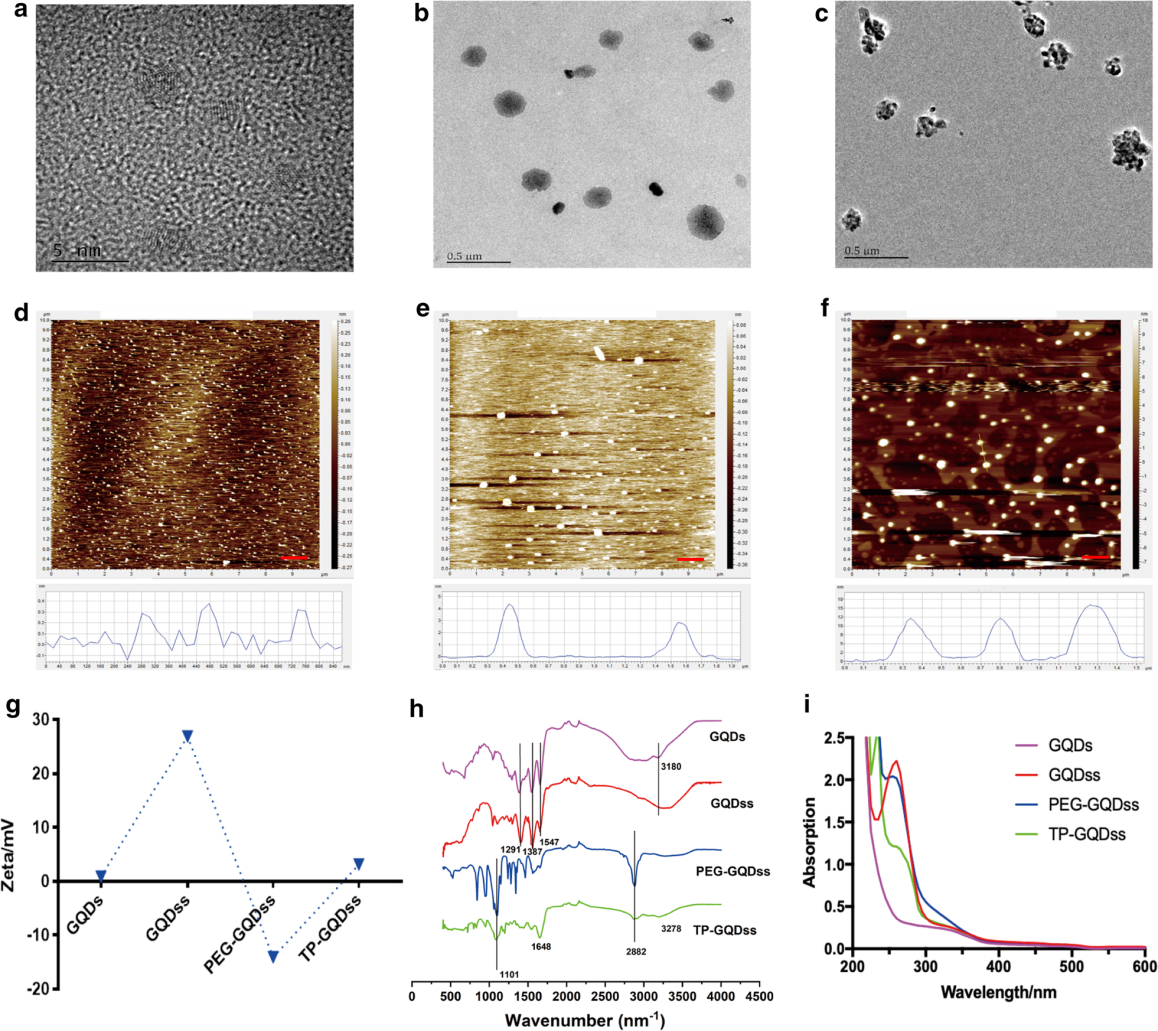
The morphologies and characterization of various GO derivatives. **a**–**c** TEM images of GQDs (scale bar = 5 nm), GQDss and TP-GQDss (scale bars = 0.5 μm); **d**–**f** AFM images of GQDs, GQDss and TP-GQDss (scale bars = 1 μm); **g** zeta potential of GQDs, GQDss, PEG-GQDss, TP-GQDss, TP-GQDss; **h** FT-IR spectra of GQDs, GQDss, PEG-GQDss, TP-GQDss; **i** UV absorption spectra of GQDs, GQDss, PEG-GQDss, TP-GQDss

The characterization results were shown in Fig. 2. As shown in Fig. 2a–c uniform circular structure was observed in the TEM images of GQDss and TP-GQDss. The size of GQDss was significantly increased from approximately 2 nm to 200 nm due to the disulfide cross-linked association of GQDs. Instead of being uniform, GQDs were polydisperse in terms of size and shape. The morphologies of various GO conjugates were further analyzed by AFM (Fig. 2d–f), which confirmed that the diameters of GQDss and TP-GQDss were approximately 200 nm. After cross-linking by disulfide bonds, the thickness of GQDs increased from 0.4 to 4 nm (GQDss) and reached 16 nm (TP-GQDss) after further modification of TP.

The zeta potential profiles of GO conjugates were shown in Fig. 2g. The zeta potential of GQDs increased from 0.8 to 26.9 mV (GQDss) after cross-linking by DTSSP, resulting from the aggregation of positive charge. An obvious reduction in zeta potential was observed in PEG-GQDss (− 14.1 mV), which may be due to the negatively charged groups in MAL-PEG-SCM. After conjugation with TP, the zeta potential of TP-GQDss returned to a positive charge (2.9 mV).

The modification of TP-GQDss was characterized by FT-IR and UV spectra analysis (Fig. 2h, i). As shown in Fig. 2h, the disappearance of typical alkyl N–H stretching frequencies at 3180 cm^−1^ demonstrated the complete removal of surfactant from GQDs, which may be due to the decreased number of amino groups in GQDss. Moreover, the appearance of O–H stretching frequencies and bending frequencies at 2882 cm^−1^ and 1101 cm^−1^, respectively, indicated that PEG-GQDss was obtained by conjugation of PEG to GQDss. Further, the FT-IR spectra also confirmed the successful synthesis of TP-GQDss by the appearance of N–H bending frequencies at 1848 cm^−1^. Additionally, Fig. 2i showed the UV absorption results of GQDs derivatives. When GQDs were cross-linked to obtain GQDss, a strong absorption appeared at 250 nm due to π–π* transition of aromatic C=C bonds and a shoulder around 300 nm due to n-π* transition of C=O bonds. After modified with PEG, the absorbance of PEG-GQDss at 320 nm increased with a slightly red shift, and the absorption of TP-GQDss, the carboxylated GQDss, was further red shift at 265 nm and 330 nm [21, 22].

### Drug release and cellular uptake of TP-GQDss

**Fig. 3:**
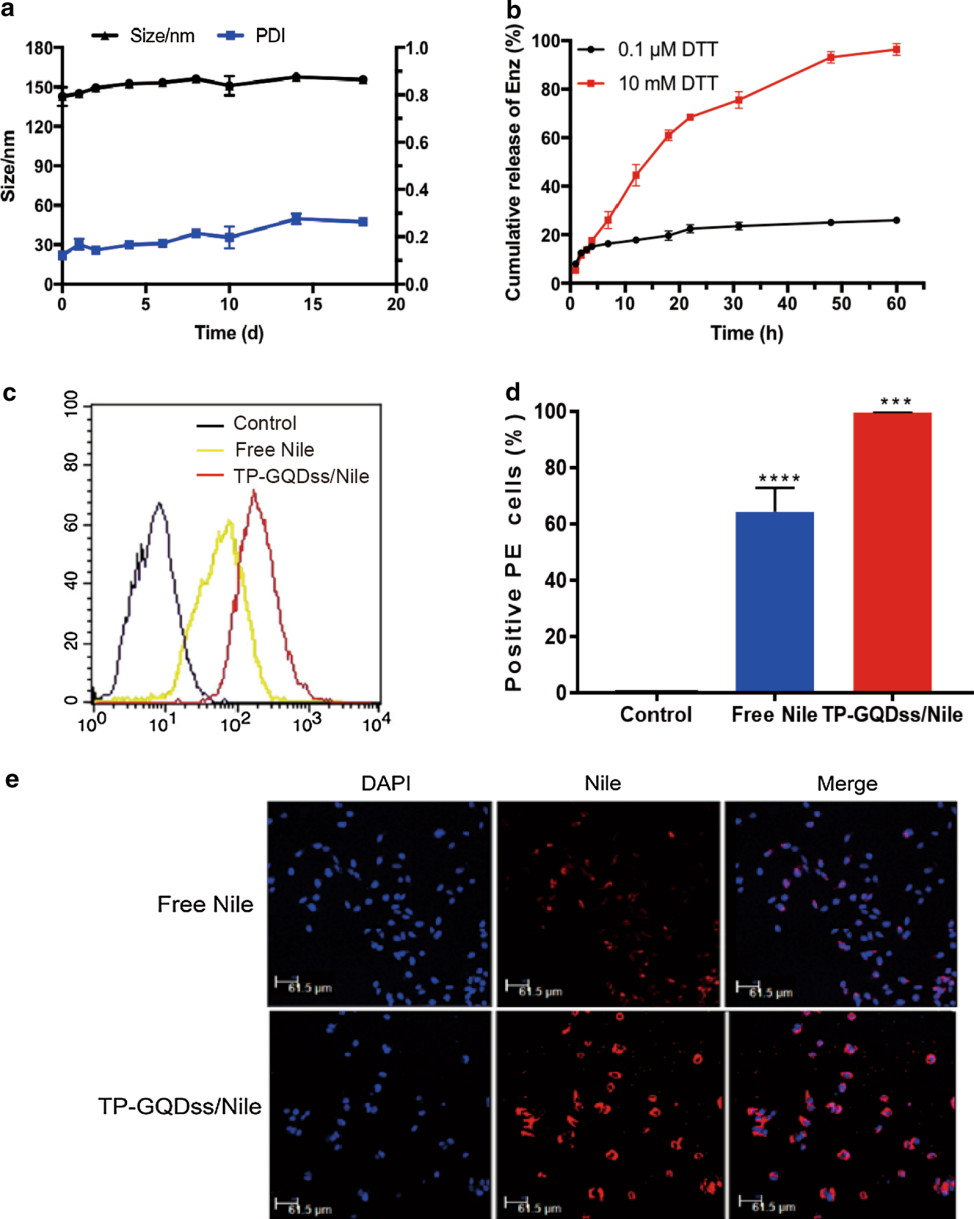
In vitro behavior of TP-GQDss. **a** Stability of TP-GQDss in PBS for 18 days, mean ± SD (n = 3); **b** drug release behavior of Enz from TP-GQDss in 0.1 μM or 10 mM DTT at pH 5.0, mean ± SD (n = 3); **c**, **d** flow cytometry results of fluorescence intensity and statistics of each group in C4-2B cells, mean ± SD (n = 3), ***p < 0.001, ****p < 0.0001; E) CLSM images of C4-2B cells following 4 h incubation with free Nile, TP-GQDss/Nile, (Nile: 20 ng/mL, scale bars = 61.5 μm)

Figure 3a showed that TP-GQDss was stable in PBS after 18 days at RT. Enz could be loaded onto TP-GQDss via π-π electron absorption, and the DL and EE of TP-GQDss reached 60% and 98%, respectively. To investigate the controlled-release efficiency of TP-GQDss under endosomal and physiological conditions, the release profiles of Enz from TP-GQDss were determined via dialysis in PBS at pH 5.0 containing 0.1 μM or 10 mM DTT (Fig. 3b). The cumulative release of Enz was only 25% with 0.1 μM DTT and reached 95% with 10 μM DTT at 48 h. The significant difference may contribute to the S–S linked structure of TP-GQDss.

The cellular uptake efficiency of Nile Red (Nile) mediated by TP-GQDss was analyzed by flow cytometry. The cells were treated with Nile and TP-GQDss/Nile for 2 h. As shown in Fig. 3c, d, an obvious increase in Nile staining was seen in TP-GQDss/Nile compared with that in the free Nile group. This indicated that the cellular uptake efficiency of Nile was significantly enhanced after incorporation into TP-GQDss. Moreover, the confocal laser scanning microscopy (CLSM) results further proved that Nile could effectively internalize into CRPC cells via TP-GQDss (Fig. 3e). No red fluorescence was observed corresponding to Nile in the nucleus of C4-2B cells, indicating that Nile was released in the cytoplasm.

### In vitro anti-proliferation abilities of TP-GQDss/Enz

**Fig. 4:**
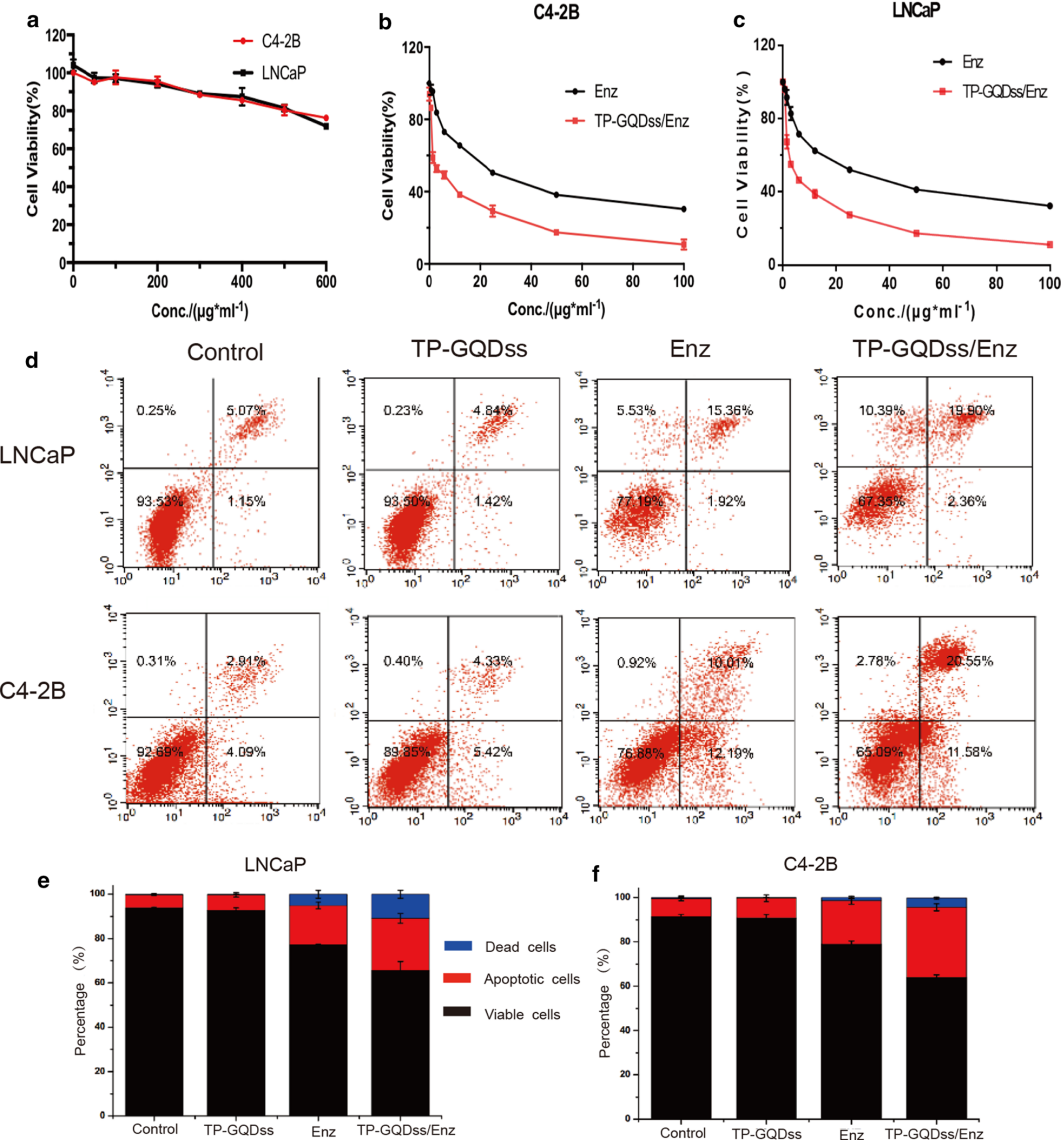
Cytotoxicity and anti-proliferation abilities of TP-GQDss, Enz and TP-GQDss/Enz. **a** Cytotoxicity of TP-GQDss in C4-2B and LNCaP cells in 48 h, mean ± SD (n = 3); **b**, **c** dose dependent in vitro anti-proliferation effect of TP-GQDss/Enz in C4-2B and LNCaP cells for 48 h, mean ± SD (n = 3); **d** apoptosis analysis of LNCaP and C4-2B cells treated with different formulations for 24 h; **e**, **f** Quantitative analysis of cell apoptosis in C4-2B and LNCaP cells, mean ± SD (n = 3)

Figure 4 showed the antiproliferation ability of TP-GQDss/Enz. As shown in Fig. 4a, the blank TP-GQDss was biocompatible at concentrations up to 400 μg/mL in either C4-2B or LNCaP cells. Furthermore, the in vitro cytotoxicity of free Enz and TP-GQDss/Enz in C4-2B and LNCaP cells was shown in Fig. 4b, c. The IC50 values of Enz and TP-GQDss/Enz were 13.5 and 3.7 μg/mL in C4-2B cells, respectively, while the IC50 values of Enz and TP-GQDss/Enz were 10.8 and 3.4 μg/mL in LNCaP cells, respectively, indicating that the cytotoxicity of Enz in prostate cancer cells was significantly enhanced by TP-GQDss.

Moreover, the apoptosis of C4-2B and LNCaP cells induced by TP-GQDss/Enz was analyzed by the Annexin V-APC/PI Apoptosis Detection Kit (Fig. 4d–f). There was no obvious difference between the negative control and the TP-GQDss (p > 0.05). The apoptosis rate in the TP-GQDss/Enz groups was much higher than that in the ENZ groups (1.5- to 2-fold), which was in line with the results of the cytotoxicity assay.

### In vivo biodistribution and effects of TP-GQDss/Enz

**Fig. 5:**
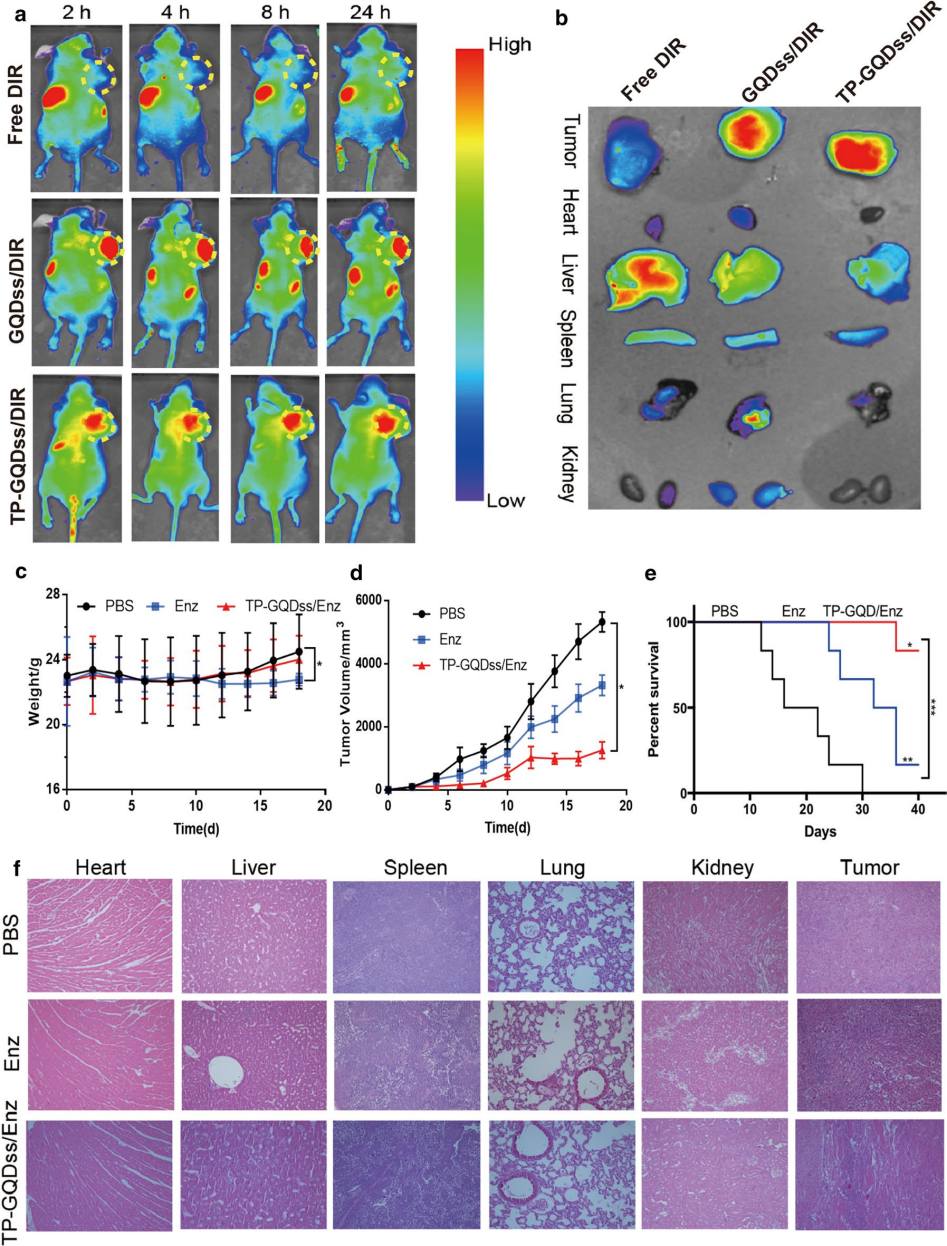
In vivo biodistribution and anti-tumour effects of TP-GQDss/Enz. **a** Fluorescent image of mice obtained at 2, 4, 8, 24 h after administration of DIR, GQDss/DIR and TP-GQDss/DIR (yellow circle: tumour site); **b** Fluorescent image of tumour and chief organs sacrificed from mice at 24 h after administration; **c**, **d** body weight variations and tumour growth curves of mice, mean ± SD (n = 6), *p < 0.05, vs PBS; **e** Survival curves of mice, log-rank analysis, mean ± SD (n = 6), *p < 0.05, **p < 0.01, ***p < 0.001; **f** Representative HE staining images of heart, liver, spleen, lung, kidney, tumour of each group (400×)

To investigate the in vivo biodistribution of TP-GQDss/Enz, a red fluorescence dye 1,1′-dioctadecyl-3,3,3′,3′-tetramethylindotricarbocyanine iodide (DIR) was used as a model drug of Enz. As shown in Fig. 5a, an obvious accumulation of DIR in the tumour was observed in both the GQDss/DIR and TP-GQDss/DIR groups at all intervals. Moreover, a DIR signal was rarely observed in any other organs in the TP-GQDss/DIR groups even until 24 h post-injection, while an obvious DIR signal was found in the lung area in the GQDss/DIR group at 2 h. The accumulation of a DIR signal in the kidney area became visible at 2 h in the GQDss/DIR group and remained stable until 24 h post-injection. In contrast, a significant DIR signal was observed in the free DIR group in the liver and kidney area at all intervals. A DIR signal was rarely found in tumour tissues or any other chief organs. In addition, tumours and chief organs of all mice were collected at 24 h post-injection for direct observation, which was in line with the in vivo imaging results (Fig. 5b). All the in vivo biodistribution results indicated that GQDss provided tumour-targeting efficiency for drug delivery, which was further enhanced via modification with a TP.

Figure 5c showed that there was no obvious difference in body weight change between the PBS and TP-GQDss/Enz groups, while the mice in the Enz group lost more weight compared with the PBS group. Moreover, significant tumour growth was observed in the free Enz group and especially in the PBS group after 10 days of treatment (Fig. 5d). The growth of tumour volume in the TP-GQDss/Enz group was strongly inhibited compared with the PBS and Enz groups. The survival time data in Fig. 5e indicated that Enz could prolong the survival of tumour-bearing mice, and TP-GQDss/Enz increased mouse survival time compared with Enz. Additionally, the HE results showed no obvious toxicity in the chief organs of each group, and the oncolysis effect of TP-GQDss/Enz was significantly higher than that of Enz (Fig. 5f).

## Discussion

Graphene oxide (GO) has shown great potential as a drug delivery system due to its sheet-like structure and π–π stacking interaction, and the drug-loading efficiency of GO is extremely high compared to other nanocarriers [23–25]. However, drug release from most GO-based nanocarriers has not been satisfactory, with less than 40% release when triggered by NIR irradiation [26]. In addition, the solubility and toxicity also restricts the application of GO as a nanocarrier [27]. A reproducible method for the preparation of nano-sized and hydrophilic GO derivatives still needs to be developed.

In this study, Enz could be effectively loaded into TP-GQDss due to the cross-linking of GQDs, with a DL of 60% and an EE of 96%, which was much higher than in the reported study [28]. In contrast with most GO derivatives, TP-GQDss/Enz could rapidly release Enz in a redox microenvironment due to that GQDss was cross-linked with disulfide bonds. Moreover, the high cell penetration and tumour-targeting efficiency of TP-GQDss was also essential to providing a suitable nano-platform for Enz. Compared with free Enz, TP-GQDss/Enz exhibited enhanced in vitro anticancer effects due to higher cellular uptake and biocompatibility, suggesting that TP-GQDss are promising nanocarriers for hydrophobic Enz delivery. In line with the in vitro results, the in vivo study also showed higher antitumour effects and better biocompatibility with TP-GQDss/Enz than with Enz. Therefore, TP-GQDss/Enz could provide a promising method, intravenous administration, for the targeted therapy of CRPC.

## Conclusion

In conclusion, we designed a redox-sensitive nano-platform (TP-GQDss) based on graphene dots with high drug-loading efficiency for intravenous delivery of Enz against CRPC. The modification of the targeting peptide and PEG on TP-GQDss contributed to good tumour-targeting efficiency and in vivo safety. Enz delivered by TP-GQDss exhibited enhanced in vitro and in vivo antitumour effects on CRPC, while the side effects of Enz were significantly reduced. Therefore, this TP-GQDss nano-platform has the potential to provide Enz-based intravenous injection therapy for CRPC.

## Methods

### Materials

Enz, aminated GQDs, MAL-PEG-SCM (PEG molecular size: 2000), l-arginine, stearic acid, and hydrogen peroxide (30% w/v) were purchased from Sangon Biotech, Shanghai, China. Dithiothreitol (DTT) was purchased from Sigma-Aldrich (St Louis, MO, USA). A luciferase assay kit was gifted from Promega (Madison, WI, USA). An enhanced bicinchoninic acid protein assay kit was purchased from Beyotime (Nanjing, China). Dulbecco’s Modified Eagle Medium (DMEM), RPMI-1640 medium, foetal bovine serum (FBS), penicillin–streptomycin solution (5 KU/mL) was obtained from Life Technologies (Carlsbad, CA, USA). Cell Counting Kit-8 (CCK-8) was purchased from Dojindo Molecular Technologies Inc., Nanjing, China. LNCaP and C4-2B cells were obtained from Shanghai Cell Bank, Chinese Academy of Sciences, Shanghai, China. All other reagents were analytical grade. All animals were supplied from Department of Pharmacy of Fudan University and all animal experiments were performed in accordance with the ethics and regulations of animal experiments at Fudan University (Shanghai, China).

### Synthesis of targeting peptide

Targeting peptide (TP, sequence: CKQFSALPFNFYT) was synthesized using the Fmoc-solid-phase peptide synthesis method by Ontores Biotech, Zhejiang, China. Briefly, 10 g of Fmoc-His(Trt)-CTC Resin was added to a medium reactor and then immersed in *N*,*N*-dimethylformamide (DMF) for 30 min. The solvent was dried down by blowing with N_2_ to remove Fmoc. Amino acid (AA), O-benzotriazole-*N*,*N*,*N′*,*N′*-tetramethyl-uronium-hexafluorophosphate (HBTU), and *N*-methylmorpholine (NMM) were added in the proportion of 3:2.85:6 to DMF. Then, the previous steps were repeated until the sequence was completed. Finally, the product was purified with High Performance Liquid Chromatography (HPLC) method to obtain 95% purity TP. The determination and purity was analyzed by MS and HPLC (Additional file 1: Figs. S1, S2).

### Synthesis and preparation of GQDss

To prepare GQDss, 1 mg/mL GQDs (XFNano, Nanjing, China) was dispersed in double distilled (DD) water or PBS, stirred at room temperature (RT). 10 mg 3,3′-dithiobis(sulfosuccinimidyl propionate) (DTSSP, Thermo Fisher, USA) was dispersed in 10 mL DD water or PBS and dropped in GQDs solution, stirred for overnight. Afterward, the GQDss solution was dialyzed against DD water (3500 MWCO) to remove free GQDs and DTSSP.

### Synthesis and preparation of TP-GQDss

To increase biocompatibility and hydrophilicity, we introduced the MAL-PEG-SCM moiety to GQDss with carboxyl groups via the catalyst *N*-(3-dimethylaminopropyl-*N′* -ethyl carbodiimide) hydrochloride (EDC). In brief, 1 mg GQDss (0.5 mg/mL in DD water) was mixed with 10 mg of MAL-PEG-SCM at RT under magnetic stirring. Then, 10 mg EDC was added, and the mixture was stirred vigorously for 24 h. Then, the obtained GQDss derivative was sonicated for 30 min. Afterward, the solution was dialyzed against DD water (3500 MWCO) to remove the excess MAL-PEG-SCM and further lyophilized. Then, PEG-functionalized GQDss (PEG-GQDss) was conjugated with TP peptide to obtain a PEG and TP peptide-conjugated multifunctional GO nanocarrier (TP-GQDss) by condensation of the MAL (of PEG) and thiol groups of cysteine of TP. PEG-GQDss was mixed with TP peptide at a molar ratio of 1:5 at pH 7.5 in the presence of Tris(2-carboxyethyl)phosphine (TCEP) to protect the thiol group from oxidation. After the overnight reaction at 4℃, the final product was dialyzed against water (3500 MWCO) to remove the excess TCEP. GQDs, GQDss, PEG-GQDss, and TP-GQDss were lyophilized for FT-IR analysis.

### Characterization of materials

The zeta potential of GQDs, GQDss, PEG-GQDss and TP-GQDss (1 mg/mL) was measured by a dynamic light scattering system (DLS) (Zetasizer Nano ZS90, Malvern) at RT. The morphologies of GQDs, GQDss and TP-GQDss were further evaluated by a JEM-1400Plus transmission electron microscope (TEM) and Bruker Dimension ICON atomic force microscope (AFM). The ultraviolet (UV) spectra and Fourier transform infrared (FT-IR) spectra were carried out to verify each step of preparation of the TP-GQDss. The UV absorption was determined using a Shimadzu UV-2450 spectrophotometer. The FT-IR spectra were recorded on an FT-IR spectrometer by compressing the samples into pellets with KBr.

### Drug loading (DL) and encapsulation efficiency (EE)

To prepare TP-GQDss/Enz, TP-GQDss aqueous solution (1 mg/mL, PBS) was mixed with Enz methanol solution (3 mM) and then stirred overnight at pH 8 [29]. The methanol was removed via dialysis (10,000 MWCO) against water for 24 h. The excessive Enz was removed by ultrafiltration (100 kDa) at 3500 rpm for 10 min with repeated rinsing in PBS (pH = 7.4, 0.01 M), at which the TP-GQDss/Enz would not separate. For DL and EE evaluation, TP-GQDss/Enz was first diluted with tenfold acetonitrile and then disrupted with ultrasonication for 20 min. DL and EE were then analyzed at 254 nm via a UV system. The formulas for DL and EE are described as follows.

$${\text{DL}}\; = \;\frac{{{\text{Enz~}}\;{\text{encapsulated~}}\;{\text{in~}}\;{\text{micelles}}}}{{{\text{Total~}}\;{\text{weight~}}\;{\text{of~}}\;{\text{TP}}\; - \;{\text{GQDss}}}}\; \times \;{\text{1}}00\%$$

$${\text{EE}}\; = \;\frac{{{\text{Enz~}}\,{\text{encapsulated~}}\;{\text{in~}}\;{\text{micelles}}}}{{{\text{Total}}\;{\text{~mount}}\;{\text{~of}}\;{\text{~Enz}}}}\; \times \;{\text{1}}00\%$$

### Drug release profiles

The drug release profiles of Enz from TP-GQDss/Enz were evaluated using a dialysis membrane method [30, 31]. TP-GQDss/Enz (1 mL, Enz: 0.5 mg/mL) was placed in a 2000 MWCO dialysis bag and immersed with 50 mL of release medium at RT. To simulate the physiological or endosomal environment where our formulations will be situated, TP-GQDss/Enz was immersed in 50 mL of PBS (50 mM) containing 0.1% (w/v) Tween 80 (pH 5.0 with 0.1 µM or 10 mM dithiothreitol (DTT)) with constant stirring at RT to simulate high concentration of glutathione in vivo. Samples of 0.2 mL volume were periodically removed and the same volume of fresh medium was added. The drug release study was performed in triplicate, and the amount of cumulative release of Enz was analyzed by a UV system.

### Cell culture and animal model

AR-positive C4-2B and LNCaP cell lines were cultured in RPMI-1640 medium with 10% FBS and 100 U/mL penicillin/streptomycin at 37 ℃ with 5% CO_2_. The C4-2B prostate cancer experimental CRPC model was established by subcutaneous injection of 2 × 10^5^ C4-2B cells suspended in PBS into 4-week-old male BALB/c nude mice (18–22 g). The mice were kept at RT, and the relative humidity was maintained in the range of 35–50%. An in vivo assay was performed after the tumour reached approximately 100 mm^3^.

### Cellular uptake assay

Nile, a hydrophobic fluorescent agent, was selected as the Enz analogue to be entrapped in TP-GQDss for intracellular uptake and CLSM. TP-GQDss/Nile was prepared using the same method as that for TP-GQDss/Enz. The drug loading efficiency of TP-GQDss/Nile was then measured by a fluorospectrophotometer.

The cell penetration efficiency of TP-GQDss was investigated by flow cytometry analysis (BD FACSCalibour, USA). Cells were seeded into 12-well plates at a density of 1.5 × 10^5^ cells/well and incubated overnight. After 24 h, the complete medium was replaced by fresh medium supplemented with TP-GQDss/Nile or Nile (20 ng/mL) and incubated for 4 h. The cells were detached with trypsin and then suspended in PBS. The results of the cellular uptake assay were determined by flow cytometry. The intracellular distribution of TP-GQDss was detected with CLSM by Nile fluorescence. Cells were seeded into 24-well plates at a density of 4 × 10^4^ cells/well and incubated overnight, and then 20 ng/mL TP-GQDss/Nile or Nile was added and incubated at RT for 4 h. After removing the medium, the cells were washed with PBS and fixed with 4% paraformaldehyde, followed by treatment with DAPI and analysis with an Olympus FV1000 confocal microscope (Center Valley, PA).

### In vitro cytotoxicity and cell apoptosis

The cytotoxicity of TP-GQDss/Enz and blank TP-GQDss was assessed in C4-2B and LNCaP cells via CCK-8 assay. Cells were seeded into 96-well plates at a density of 8 × 10^3^ cells/well and incubated overnight. After 24 h, the complete medium was replaced by fresh medium containing TP-GQDss (0–600 μg/mL), TP-GQDss/Enz (Enz: 0–100 μg/mL) or free Enz (0–100 μg/mL) and incubated for an additional 24 h. Free Enz was dissolved in a solution (DMSO: polyethylene glycol 400: PBS = 10: 45: 45, v/v/v) [5]. CCK-8 (10 μL, 5 mg/mL) was added into each well and incubated for 2 h to measure the absorbance of each group at 450 nm.

For cell apoptosis analysis, cells were seeded into 12-well plates at a density of 1.5 × 10^5^ cells/well overnight. After 24 h, the complete medium was replaced by fresh medium with TP-GQDss/Enz (Enz: 10 μg/mL) and incubated for an additional 48 h. Free Enz (10 μg/mL) and TP-GQDss (50 μg/mL) were used as controls. After 48 h of incubation, the cells were detached with trypsin, suspended in PBS and stained with Annexin V-APC/PI. The results of the cell apoptosis assay were determined by flow cytometry (FACS Calibur; BD Biosciences, UK).

### In vivo distribution

A near-infrared fluorescence substance DIR was entrapped in TP-GQDss for the in vivo biodistribution assay. TP-GQDss/DIR was prepared using the same method as TP-GQDss/Enz. Solid tumours were induced in nude mice by subcutaneously injecting C4-2B cells (5 × 10^6^ cells/nude mouse) into the right front limb. Animals were acclimated to laboratory conditions for 1 week. When tumour volumes grew to 150 mm^3^, the tumour-bearing nude mice were divided into 3 groups (3 mice per group) randomly. All the mice were injected with free DIR, GQDss/DIR and TP-GQDss/DIR by tail vein injection respectively and scanned at 2, 6, 12 and 24 h post-injection using a fluorescence imaging system. Animals were sacrificed at 24 h to excise tumours, hearts, livers, spleens, lungs and kidneys, and the fluorescence intensity in each tissue or organ was measured using an in vivo imaging system.

### In vivo antitumour effects

C4-2B cells (1 × 10^6^) were suspended in 100 μL of PBS and injected into the right front limb subcutaneously of male 6-week-old BALB/c nude mice. The mice were divided into three groups (6 mice per group) and injected with PBS, Enz and TP-GQDss/Enz by tail vein injection when the average tumour volume reached approximately 100 mm^3^ (Enz equivalent: 20 mg/kg, n = 6). All the mice were administrated every 2 days for 6 times. Tumour size was measured every two days using a Vernier caliper. Tumour volume (V) was calculated by the following formula: V (mm^3^) = width^2^ × length/2. The weight of mice was also recorded to assess safety. Additionally, mice in the PBS, Enz and TP-GQDss/Enz groups were sacrificed at the 40th day after tumor induction, and the tumours, hearts, livers, spleens, lungs and kidneys were dissected for haematoxylin–eosin (HE) staining.

### Statistical analysis

All values are presented as the mean ± SD. Statistical significance was determined using ANOVA and log-rank analysis. p < 0.05 was considered statistically significant.

## Supplementary information

**Additional file 1: Fig. S1.** MS determination of TP. **Fig. S2.** The result of TP purity determined by HPLC.

## Data Availability

All data generated or analyzed during this study are included in this published article.

## Abbreviations

Enz: Enzalutamide
CRPC: Castration-resistant prostate cancer
GQDs: Graphene quantum dots
TP: Targeting peptide
ADT: Androgen deprivation therapy
AR: Androgen receptor
GO: Graphene oxide
NIR: Near-infrared radiation
GQD-NH_2_: Aminated graphene quantum dots
DTSSP: 3,3′-Dithiobis(sulfosuccinimidyl propionate)
CCK-8: Cell counting kit-8
DMF: *N*,*N*-Dimethylformamide
AA: Amino acid
HBTU: *O*-Benzotriazole-*N*,*N*,*N*′,*N*′-tetramethyl-uronium-hexafluorophosphate
NMM: *N*-Methylmorpholine
EDC: *N*-(3-Dimethylaminopropyl-*N*′-ethyl carbodiimide) hydrochloride
DD: Double distilled
RT: Room temperature
DLS: Dynamic light scattering system
TEM: Transmission electron microscope
AFM: Atomic force microscope
UV: Ultraviolet
FT-IR: Fourier transform infrared
DL: Drug loading
EE: Encapsulation efficiency
DTT: Dithiothreitol
CLSM: Confocal laser scanning microscopy
Nile: Nile red
DIR: Iodide[1,1-dioctadecyl-3,3,3,3-tetramethylindotricarbocyanine iodide]
HE: Haematoxylin–eosin
SD: Standard deviation
ANOVA: Analysis of variance

## References

[1] Wallis CJ, Satkunasivam R (2017). Prostate cancer: risk factors – you find what you are looking for. Nat Rev Urol.

[2] Bianchini D, Lorente D, Rodriguez-Vida A, Omlin A, Pezaro C, Ferraldeschi R (2014). Antitumour activity of enzalutamide (MDV3100) in patients with metastatic castration-resistant prostate cancer (CRPC) pre-treated with docetaxel and abiraterone. Eur J Cancer.

[3] Kim TH, Jeong JW, Song JH, Lee KR, Ahn S, Ahn SH (2015). Pharmacokinetics of enzalutamide, an anti-prostate cancer drug, in rats. Arch Pharm Res.

[4] Krauwinkel W, Noukens J, Van Dijk J, Popa S, Ouatas T, De Vries M (2017). A comparison of the pharmacokinetics and safety of enzalutamide in subjects with hepatic impairment and matched healthy subjects. J Clin Pharm Ther.

[5] Crona DJ, Milowsky MI, Whang YE (2015). Androgen receptor targeting drugs in castration-resistant prostate cancer and mechanisms of resistance. Clin Pharmacol Ther.

[6] Scher HI, Beer TM, Higano CS, Anand A, Taplin ME, Efstathiou E, Rathkopf D, Shelkey J (2010). Antitumour activity of MDV3100 in castration-resistant prostate cancer: a phase 1–2 study. Lancet.

[7] Cheng CJ, Tietjen GT, Saucier-Sawyer JK, Saltzman WM (2015). A holistic approach to targeting disease with polymeric nanoparticles. Nat Rev Drug Discov.

[8] Chen J, Wu Z, Ding W, Xiao C, Zhang Y, Gao S (2019). SREBP1 siRNA enhance the effect of docetaxel based on a bone-cancer dual-targeting biomimetic nanosystem against bone metastatic castration-resistant prostate cancer. Theranostics.

[9] Qiang L, Cai Z, Jiang W, Liu J, Tai Z, Li G (2019). A novel macrophage-mediated biomimetic delivery system with NIR-triggered release for prostate cancer therapy. J Nanobiotechnology.

[10] Xia Q, Gong C, Gu F, Wang Z, Hu C, Zhang L (2018). Functionalized multi-walled carbon nanotubes for targeting delivery of immunostimulatory CpG oligonucleotides against prostate cancer. J Biomed Nanotechnol.

[11] Feeney OM, Crum MF, McEvoy CL, Trevaskis NL, Williams HD, Pouton CW (2016). 50 years of oral lipid-based formulations: provenance, progress and future perspectives. Adv Drug Deliv Rev.

[12] Wang H, Sun D, Zhao N, Yang X, Shi Y, Li J (2014). Thermo-sensitive graphene oxide-polymer nanoparticle hybrids: synthesis, characterization, biocompatibility and drug delivery. J Mater Chem B.

[13] Xu Z, Zhu S, Wang M, Li Y, Shi P, Huang X (2015). Delivery of paclitaxel using PEGylated graphene oxide as a nanocarrier. ACS Appl Mater Inter.

[14] Yang K, Zhang S, Zhang G, Sun X, Lee ST, Liu Z (2010). Graphene in mice: ultrahigh in vivo tumor uptake and efficient photothermal therapy. Nano Lett.

[15] Li H, Flerens K, Zhang Z, Vanparijs N, Schuijs MJ, Van Steendam K (2016). Spontaneous protein adsorption on graphene oxide nanosheets allowing efficient intracellular vaccine protein delivery. ACS Appl Mater Inter.

[16] Li J, Liang X, Zhang J, Yin Y, Zuo T, Wang Y (2018). Inhibiting pulmonary metastasis of breast cancer based on dual-targeting graphene oxide with high stability and drug loading capacity. Nanomedicine.

[17] Zhang X, Gong C, Akakuru OU, Su Z, Wu A, Wei G (2019). The design and biomedical applications of self-assembled two-dimensional organic biomaterials. Chem Soc Rev.

[18] Song E, Han W, Li C, Cheng D, Li L, Liu L (2014). Hyaluronic acid-decorated graphene oxide nanohybrids as nanocarriers for targeted and pH-responsive anticancer drug delivery. ACS Appl Mater Inter.

[19] Pan Y, Sahoo NG, Li L (2012). The application of graphene oxide in drug delivery. Expert Opin Drug Deliv.

[20] Yeh CY, Hsiao JK, Wang YP, Lan CH, Wu HC (2016). Peptide-conjugated nanoparticles for targeted imaging and therapy of prostate cancer. Biomaterials.

[21] Novak TG, Kim J, Song SH, Jun GH, Kim H, Jeong MS (2016). Fast P_3_HT exciton dissociation and absorption enhancement of organic solar cells by PEG-functionalized graphene quantum dots. Small.

[22] Liang RP, Qiu WB, Zhao HF, Xiang CY, Qiu JD (2016). Electrochemiluminescence resonance energy transfer between graphene quantum dots and graphene oxide for sensitive protein kinase and inhibitor sensing. Anal Chim Acta.

[23] Yu X, Cheng H, Zhang M, Zhao Y, Qu L, Shi G (2017). Graphene-based smart materials. Nat Rev Mater.

[24] Shim G, Kim MG, Park JY, Oh YK (2016). Graphene-based nanosheets for delivery of chemotherapeutics and biological drugs. Adv Drug Deliv Rev.

[25] Zhao H, Ding R, Zhao X, Li Y, Qu L, Pei H (2017). Graphene-based nanomaterials for drug and/or gene delivery, bioimaging, and tissue engineering. Drug Discov Today.

[26] Tran TH, Nguyen HT, Pham TT, Chol JY, Choi HG, Yong CS (2015). Development of a graphene oxide nanocarrier for dual-drug chemo-phototherapy to overcome drug resistance in cancer. ACS Appl Mater Inter.

[27] Yang K, Feng L, Liu Z (2015). The advancing uses of nano-graphene in drug delivery. Expert Opin Drug Deliv.

[28] Thangavel C, Perepelyuk M, Boopathi E, Liu Y, Polischak S, Deshpande DA (2018). Improvement in therapeutic efficacy and reduction in cellular toxicity: introduction of a novel anti-PSMA-conjugated hybrid antiandrogen nanoparticle. Mol Pharm.

[29] Yang D, Feng L, Dougherty CA, Luker KE, Chen D, Cauble MA (2016). In vivo targeting of metastatic breast cancer via tumor vasculature-specific nano-graphene oxide. Biomaterials.

[30] Nasrollahi F, Varshosaz J, Khodadadi AA, Lim S, Jahanian-Najafabadi A (2016). Targeted delivery of docetaxel using transferrin/poly(allylaminehydrochloride)-functionalized graphene oxide nanocarrier. ACS Appl Mater Interfaces.

[31] Tian Y, Guo R, Jiao Y, Sun Y, Shen S, Wang Y (2016). Redox stimuli-responsive hollow mesoporous silica nanocarriers for targeted drug delivery in cancer therapy. Nanoscale Horiz.

