# Supplementary material for: Intravenous delivery of enzalutamide based on high drug loading multifunctional graphene oxide nanoparticles for castration-resistant prostate cancer therapy
Source: J Nanobiotechnology. 2020 Mar 18;18:50. doi: 10.1186/s12951-020-00607-4 (PMC7079409; doi:10.1186/s12951-020-00607-4)
Supplement: Supplementary file 1 — Additional file 1: Fig. S1. MS determination of TP. Fig. S2. The result of TP purity determined by HPLC. [file 12951_2020_607_MOESM1_ESM.docx]

Additional Information

Intravenous delivery of enzalutamide based on high drug loading multifunctional graphene oxide nanoparticles for castration-resistant prostate cancer therapy

Wenjun Jiang, ^†a,b^ Jiyuan Chen, ^†a^ Chunai Gong, ^c^ Yuanyuan Wang, ^a^ Yuan Gao, *^a,d^ Yongfang Yuan *^c^

^a^ Department of Clinical Pharmacy and Pharmaceutical Management, School of Pharmacy, Fudan University, 826 Zhangheng Road, Shanghai 201203, China.

^b^ Department of Pharmacy, East China University of Science and Technology, 130 Meilong Road, Shanghai 200237, China.

^c^ Department of Pharmacy, Shanghai Ninth People’s Hospital, Shanghai Jiao Tong University School of Medicine, 639 Zhizaoju Road, Shanghai 200011, China.

^d^ Department of Pharmacy, Changhai Hospital, Second Military Medical University, 168 Changhai Road, Shanghai 200433, China.

^†^ These authors contributed equally to this work.

*Corresponding authors. Tel./Fax: +86 21 51980176 (Yuan Gao), Tel./Fax: +86 21 63087073 (Yongfang Yuan).

E-mail addresses: [yuan_gao@fudan.edu.cn](mailto:yuan_gao@fudan.edu.cn) (Yuan Gao), [nmxyyf@126.com](mailto:nmxyyf@126.com) (Yongfang Yuan)

.
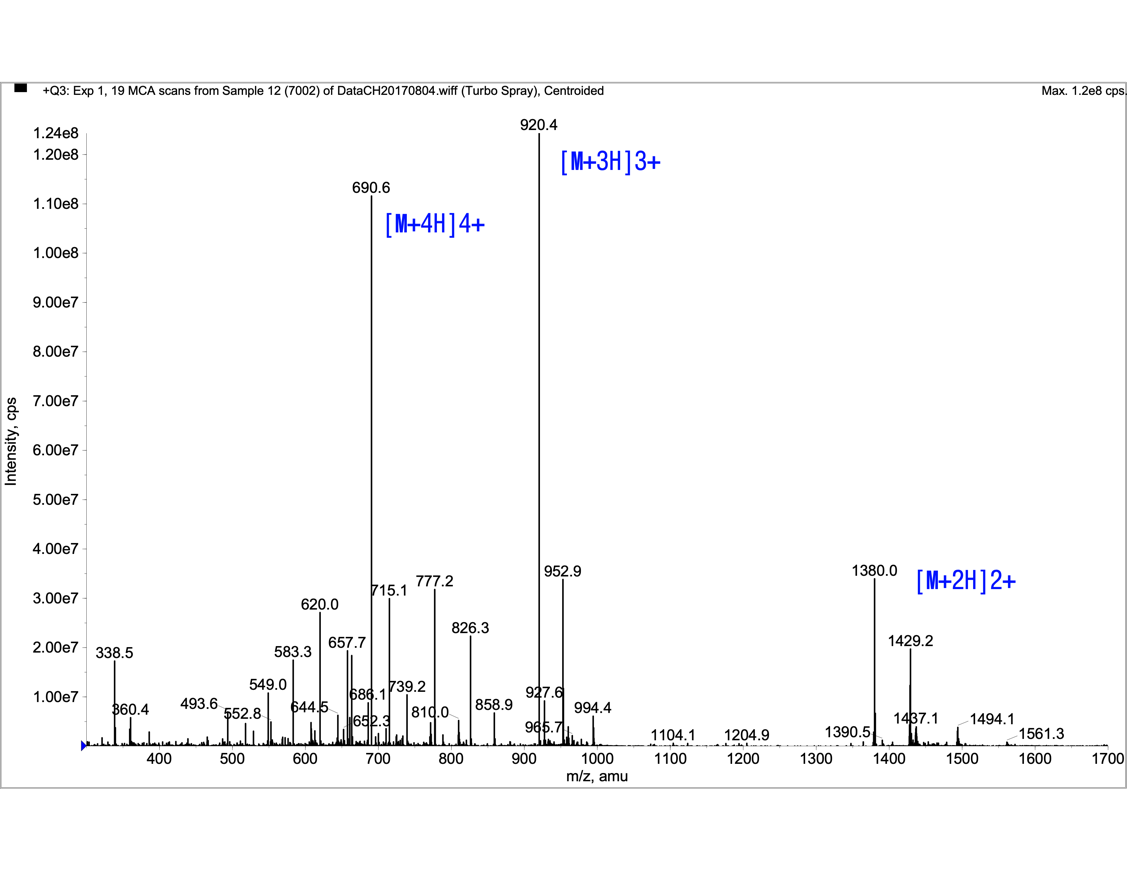


Fig. S1. MS determination of TP.


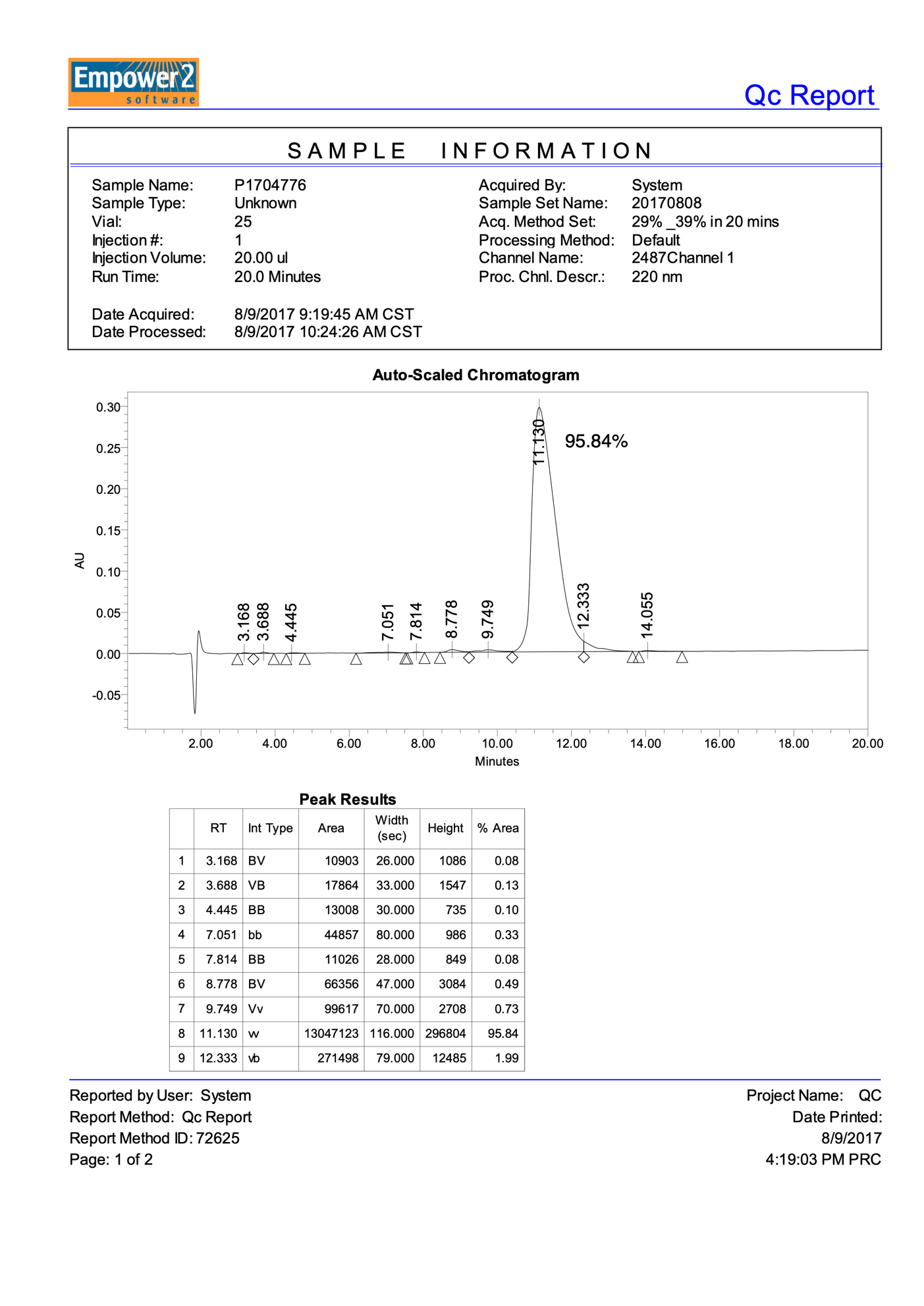


Fig. S2. The result of TP purity determined by HPLC.
